# Comparison of weight regain practices between professional MMA organizations between 2018–2023

**DOI:** 10.1080/15502783.2025.2550160

**Published:** 2025-08-22

**Authors:** Peter Byers, Savannah Calleja, Antonella Schwarz, Gabriel J. Sanders, Tobin Silver, Cassandra Evans, Tony Ricci, Corey A. Peacock, Jose Antonio

**Affiliations:** a Nova Southeastern University, Department of Health and Human Performance, Davie, FL, USA; b Barry University, Department of Sport and Exercise Sciences, Miami Shores, FL, USA; c University of Cincinnati, Exercise Science, Cincinnati, OH, USA

**Keywords:** Weigh-in, MMA, outcome, organizations

## Abstract

**Background:**

MMA fighters commonly engage in rapid weight loss (RWL) and rapid weight regain (RWRG) to gain a competitive advantage over their opponents. Fighters manipulate their body weight to compete in weight classes one to two divisions higher than their official weigh-in. Previous research has examined whether the percentage of weight regained is predictive of wins and losses in professional MMA organizations. However, little research has compared RWRG between the official weigh-in and fight night weight in professional organizations, notably the Ultimate Fighting Championship (UFC) and Bellator. Therefore, the purpose of this study is to compare RWRG between professional MMA fighters from the UFC and Bellator.

**Methods:**

This study included 245 professional MMA fighters (UFC: *n* = 89, 29.8 ± 3.9 years, 175.1 ± 9.9 cm; Bellator: *n* = 156, 30.4 ± 4.9 years, 175.6 ± 7.3 cm). All fights occurred between 2018 and 2023, with data made public by the California State Athletic Commission (CSAC). Fighters had 30–36 hours to rehydrate between the official weigh-in and fight night. Weigh-ins were supervised by the CSAC using commission-calibrated scales. Independent samples t-tests were utilized to compare the percentage of weight regained between the two organizations, with subgroup analyses by sex. Paired samples t-tests were used to compare official weigh-in and fight night weights. Statistical significance was set at *p* ≤ 0.05.

**Results:**

Fighters from both organizations significantly increased their body weight between the official weigh-in and fight night (*p* < .001). However, no significant difference was found in the percentage of body weight regained between organizations (Bellator vs. UFC, *p* = .087, *t* = 1.72). Subgroup analyses revealed that both males (*p* < .001) and females (*p* < .001) significantly increased their body weight between the official weigh-in and fight night; however, no significant difference in the percentage of weight regained was observed by sex (*p* = .523, *t* = 0.640).

**Conclusion:**

MMA fighters from the UFC and Bellator significantly increase their body weight following the official weigh-in; however, the percentage of weight regained does not differ between organizations. These findings align with existing literature on RWL and RWRG practices among MMA fighters and should help inform weight-cut specialists to ensure fighter safety. Future research could examine whether RWRG impacts outcomes in terms of wins and losses in large sample sizes for professional organizations.

